# Supplementary material for: MiR-126-Loaded Immunoliposomes against Vascular Endothelial Inflammation In Vitro and Vivo Evaluation
Source: Pharmaceutics. 2023 Apr 30;15(5):1379. doi: 10.3390/pharmaceutics15051379 (PMC10221669; doi:10.3390/pharmaceutics15051379)
Supplement: Supplementary file 1 [file pharmaceutics-15-01379-s001.zip › pharmaceutics-2243070-supplementary.pdf]

Supplementary Information

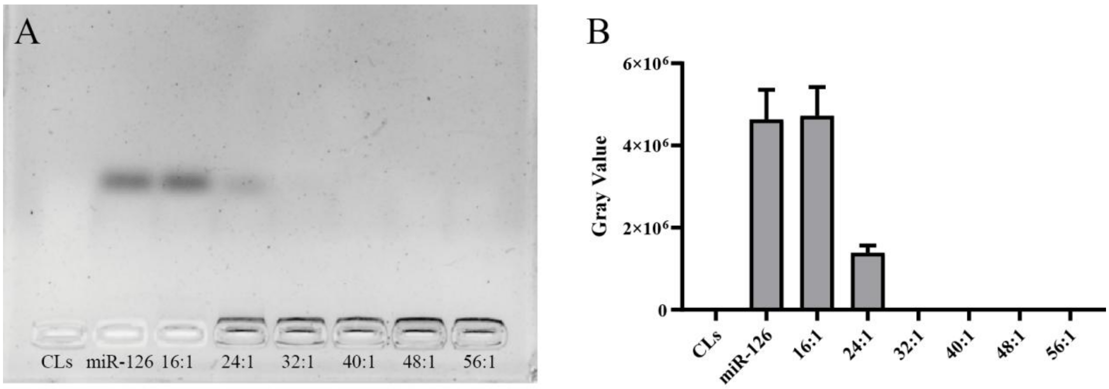

Figure S1. The results of agarose gel electrophoresis. (A) Identification of miR-126 conjugates by gel retardation assay. 1, miR-126; 2, CLs; 3-8,DOTAP:miR-126 =16:1，24:1，32:1，40:1，48:1，56:1. (B) Gray value of each miR-126 band calculated by Image J.

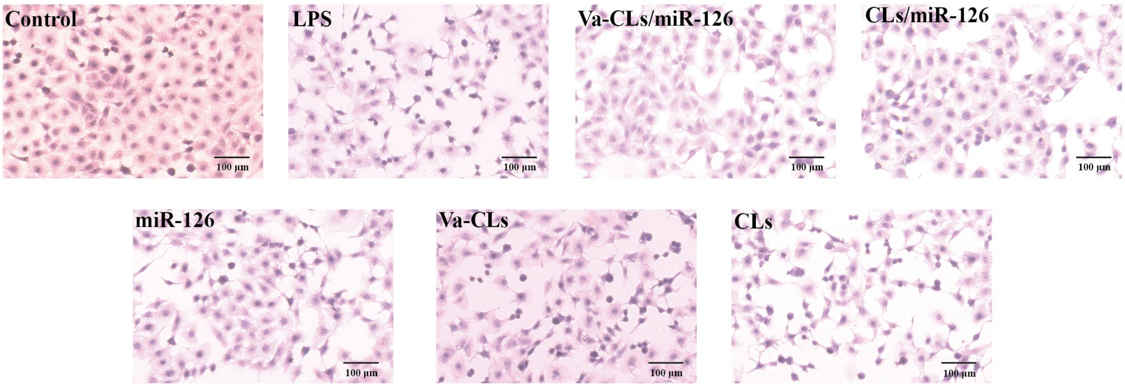

Figure S2. Effects of Va-CLs/miR-126 on morphology of HUVECs induced by LPS (10×).
